# Achromobacter xylosoxidans Subdiaphragmatic Collection as a Result of a Dropped Stone During Laparoscopic Cholecystectomy

**DOI:** 10.7759/cureus.17881

**Published:** 2021-09-10

**Authors:** Zaid A Alzeer, Mohammad A Alghafees, Khalid Bedah

**Affiliations:** 1 College of Medicine, King Saud Bin Abdulaziz University for Health Sciences, Riyadh, SAU; 2 Surgery, King Abdulaziz Medical City Riyadh, Riyadh, SAU

**Keywords:** dropped stones, laparascopic surgery, hepatobiliary surgeries, subdiaphragmatic collection, post cholecystectomy

## Abstract

After a cholecystectomy, dropped stones can serve as a nidus for abscess formation. Intrabdominal abscesses tend to cause irritation and inflammation of the peritoneum and are thus rarely asymptomatic. This report discusses a 38-year-old female complaining of a recurrent right upper quadrant pain that led to multiple hospital admissions. Her surgical history was significant for cholecystectomy six years back complicated by a retroperitoneal abscess which was drained twice. A computed tomography (CT) scan was done, and she was diagnosed with a subdiaphragmatic collection as a result of a dropped stone.

## Introduction

Laparoscopic cholecystectomy (LC), although widely accepted and frequently performed as a minimally invasive procedure, has some possible complications and known adverse events. One of the several potential complications of LC is gallstone loss and spillage. Although unappreciated, this complication is known to occur in 6% to 40% of all laparoscopic cholecystectomies [1-3]. Gallstones are often left within the intraperitoneal cavity despite surgeons’ attempts at laparoscopic removal of spilled gallstones because the stones are often inaccessible, fragmented, or left unnoticed. A small proportion of patients with dropped gallstones develops complications. One meta-analysis of eight studies on gallstones unretrieved during LC found that nearly 2% of all laparoscopic cholecystectomies result in unretrieved gallstones and 8.5% of these patients develop complications [1]. An abscess in the abdominal wall or the perihepatic space is the most frequently occurring complication of lost gallstones [4,5]. The first year post-cholecystectomy is when a post-operative abscess commonly forms. However, delayed abscess formation due to dropped gallstones may occur up to 15 years post-cholecystectomy [6]. The abscess is most commonly located in the abdominal wall but may be located in the intra-abdominal cavity, particularly in the sub-hepatic space, or the retroperitoneum inferior to the sub-hepatic space [1]. In this report, a rare case of subdiaphragmatic collection that formed six years post-laparoscopic cholecystectomy is presented.

## Case presentation

A 38-year-old female was admitted to the emergency department with worsening right upper quadrant pain for seven days, which was most severe during the first 24 hours of presentation. The pain was mainly in the right upper quadrant and on the back below the ribs, radiating to the right shoulder. Her appetite was on the lower side. Her surgical history was significant for a laparoscopic cholecystectomy approximately six years ago which was complicated by a retroperitoneal abscess and drained twice, laparoscopic appendectomy eight years ago, and four C-sections of which the last one was four months ago.

Her blood pressure (BP) was 120/88, heart rate was 79 beats per minute (bpm), respiratory rate was 20 breaths per minute, her temperature was 37.1 °C, and her oxygen saturation (spO2) was 100%. On examination, she was well-oriented without any distress. Her abdominal examination showed positive rebound tenderness in the right suprainguinal region. The rest of the examination was unremarkable.

**Figure 1:**
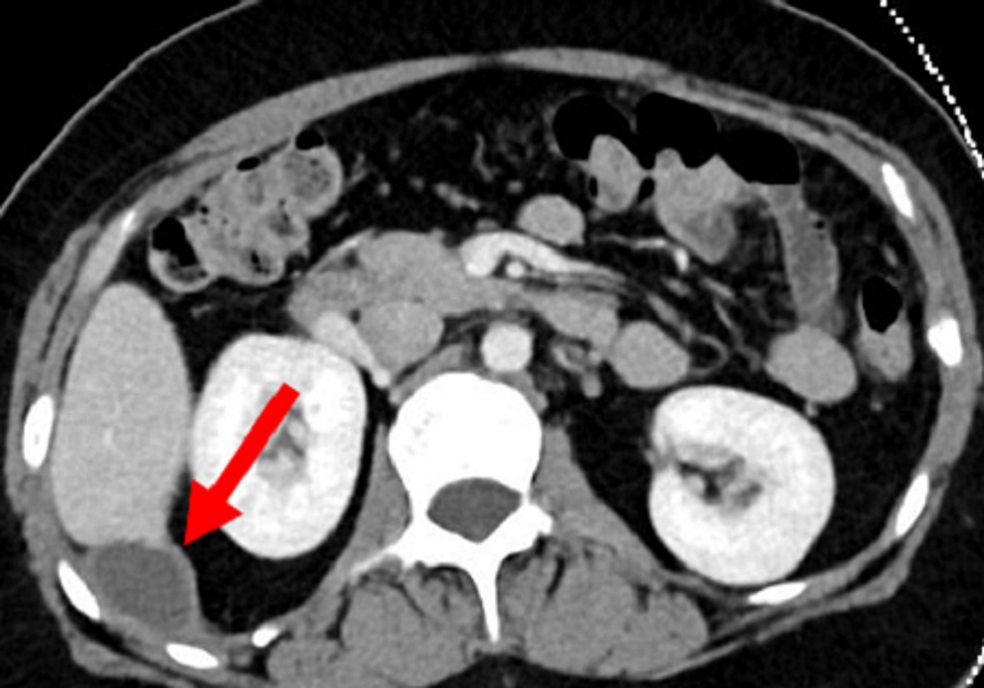
A transverse view of the CT scan showing a subdiaphragmatic collection (red arrow). CT: Computed tomography.

**Figure 2:**
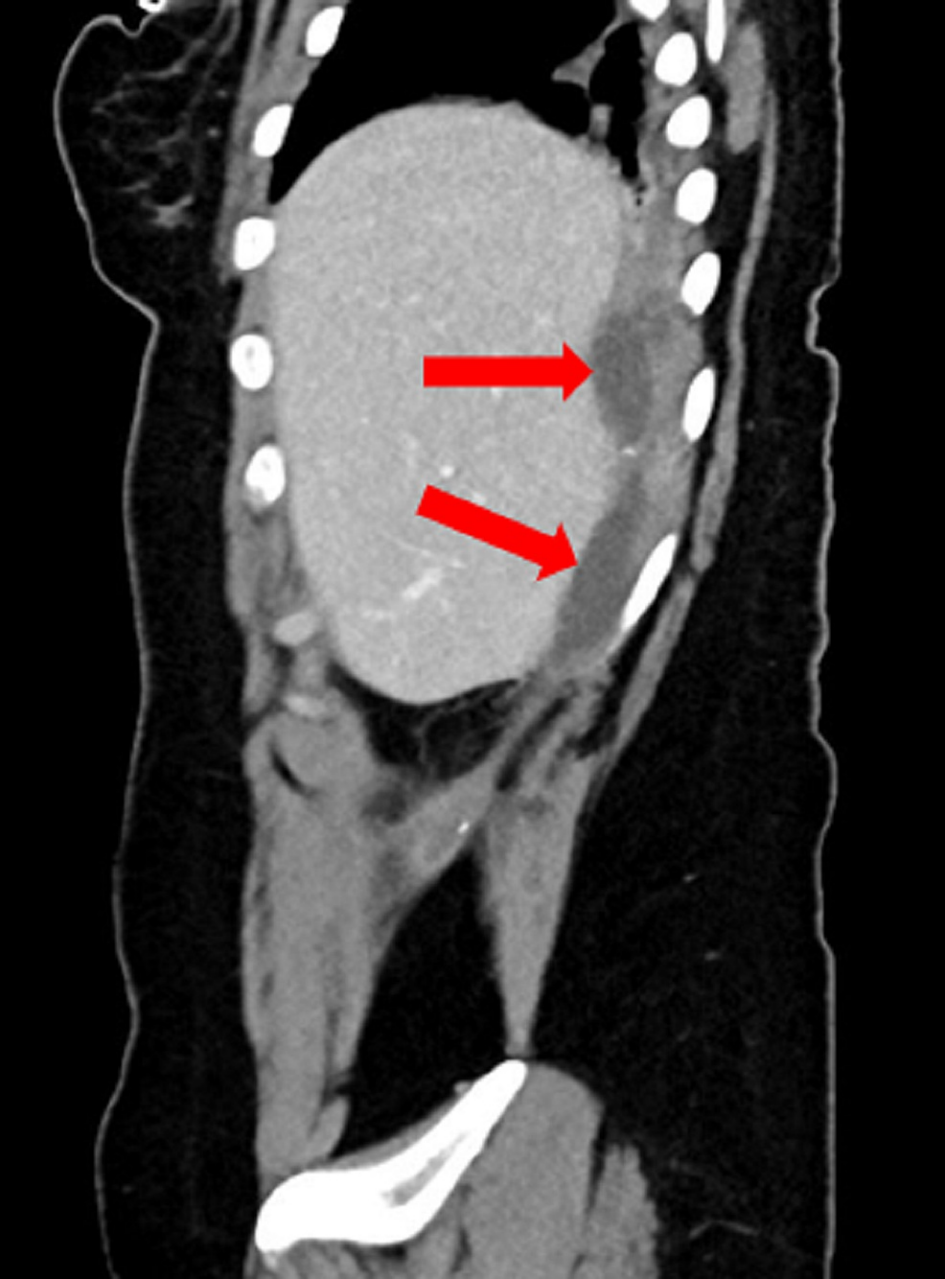
A sagittal view of the CT scan showing a subdiaphragmatic collection (red arrows). CT: Computed tomography.

Laboratory workup revealed a hemoglobin of 13.9 mg/dl, platelets of 255 × 10^9^/L, white blood cells of 10.2 × 10^9^/L, erythrocyte sedimentation rate (ESR) of 28, a lactic acid of 2.28, an amylase of 39, a bilirubin total of 2.1, alanine aminotransferase (ALT) of 22, and aspartate aminotransferase (AST) of 15. Urea and electrolytes were all within the normal limits. On X-ray, no radiopacity could have been visualized. An abdominopelvic CT scan was ordered and revealed an interval increase in the size of a subdiaphragmatic collection measuring 10x2.9x3.3 cm (Figure 1, 2). The collection had an extension to the subcapsular hepatic space and to the back muscles on the right side. A dropped stone in which was left in the index laparoscopic cholecystectomy was marked as the cause of this recurrent collection.

The patient was kept nil per os, started on intravenous fluids, and antibiotics were given. The interventional radiologist was consulted. A drain catheter was inserted, and 30 ml of pus was drained. The sample was sent for culture which showed a light growth of Achromobacter xylosoxidans. The patient’s pain improved, and she remained pain-free and afebrile during her stay. On the second day, 60 ml of pus was drained, and the drain was removed. The patient was tolerating orally, symptom-free, and discharged home on oral antibiotics. Surgical retrieval of the dropped stone was proposed, but the patient refused.

## Discussion

We report a case of a 38-year-old female patient who developed a subdiaphragmatic collection due to unretrieved gallstones. To date, only one case of subdiaphragmatic collection due to dropped gallstones has been reported in a 67-year-old male patient [7].

The gold-standard treatment for symptomatic gallstone disease is laparoscopic cholecystectomy, developed in Germany in 1985 by Erich Mühe [8]. This procedure, however, is associated with complications including injury to blood vessels, gastrointestinal tract (GIT), and biliary system, and gallbladder perforation with spillage of bile and gallstones, particularly when the wall is gangrenous or inadvertently injured by diathermy. The incidence of these complications is 6%-40% of all laparoscopic cholecystectomy operations [9,10]. Spilled gallstones complicate nearly one-third of all laparoscopic cholecystectomies. The dropping of gallstones may also occur during open cholecystectomies, but the gallstones are more easily retrieved owing to a larger operating field [11,12]. Abscess formation is the most common complication of unretrieved gallstones. Fifty-six percent of the abscesses are located intraperitoneally (commonly in the subhepatic region), 20% are in the abdominal wall, 13% in the thoracic wall, and 11% are retroperitoneal [12]. It was estimated by Lohan et al. [11] that 0.6%-2.9% of the cases of bile and gallstone spillage are produced in the peritoneal recesses.

Dropped gallstones may reach the retroperitoneum after dropping from the gallbladder by passing behind the liver (Segment VI) and right colon towards the retroperitoneum. It is noteworthy that an abscess due to spilled gallstones may be asymptomatic in some cases. If dropped gallstones are present in the retroperitoneum, back pain and abdominal pain are the most common symptoms; a fever is also seen in 25% of the patients. The median time of appearance of symptoms is one year post-cholecystectomy, but exceptional cases with symptoms appearing up to 20 years post-cholecystectomy have also been reported in the literature [1]. The best method to diagnose an abscess due to unretrieved gallstones is a thoracoabdominal CT [13].

­Infection of unretrieved gallstones, when it occurs and causes symptoms, is treated with antimicrobial therapy as well as surgical or percutaneous removal of the gallstones [14]. In this case, antibiotics were used in conjunction with the percutaneous removal of the pus.

## Conclusions

This case is unusual in terms of isolated species and management. Although the overall complications of dropped stones are uncommon, they do present a significant burden of morbidity for a long period of time when they occur. Percutaneous drainage may not be adequate as a radical treatment to dropped stones after laparoscopic cholecystectomy. The need for removing the stones becomes necessary especially when a recurrent presentation with the same collection occurs. MRI may reveal the stones that are difficult to visualize on CT scans of the abdomen. Considerable precautions should be taken to retrieve the dropped stones and to avoid gallbladder perforation during laparoscopic cholecystectomy, especially if the wall is gangrenous or in the case of injury by diathermy.
